# Supplementary figures and images for: Mature tau pathology is not improved by interfering with interleukin-1 receptor signaling in two mouse models of tauopathy
Source: PLoS One. 2025 Nov 5;20(11):e0335409. doi: 10.1371/journal.pone.0335409 (PMC12588532; doi:10.1371/journal.pone.0335409)

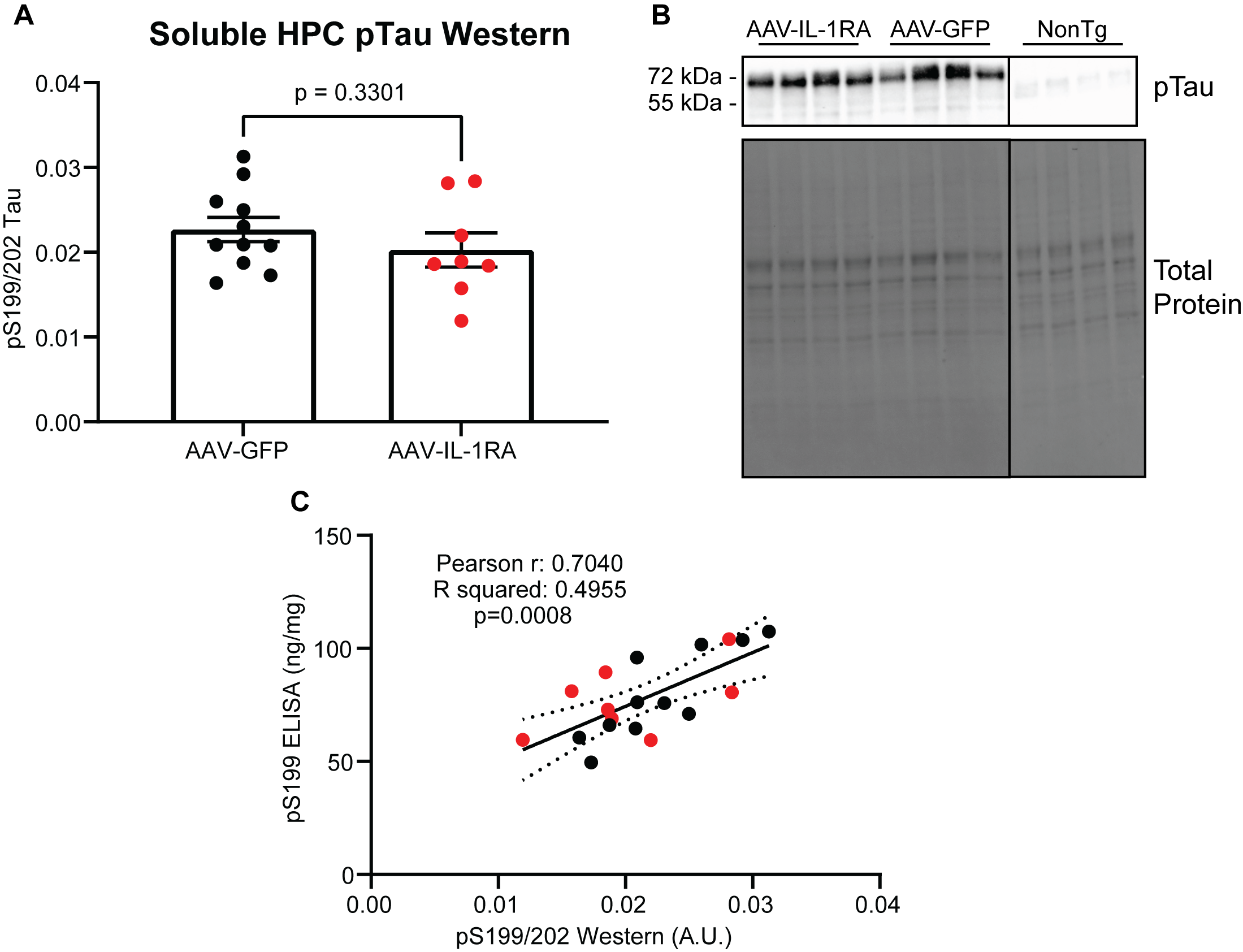

Supplement: S2 Fig — (A) Graph of pS199/202 phospho-tau detected by Western blot relative to total protein loading control. No significant difference observed between AAV-GFP and AAV-IL-1RA injected mice. Data analyzed by two-tailed Student’s t-test. (B) Representative images of pS199/202 phospho-tau (top panel) and total protein loading control (bottom panel). (C) Graph of pS199/202 phospho-tau levels by western correlated with pS199 phospho-tau levels detected by ELISA. A significant correlation was observed between the two methods (p = 0.0008). Red dots represent AAV-IL-1RA injected mice and black dots represent AAV-GFP injected mice. Data presented as mean ± SEM, n = 8–11. (TIF) [file pone.0335409.s002.tif]
