# Supplementary material for: Mature tau pathology is not improved by interfering with interleukin-1 receptor signaling in two mouse models of tauopathy
Source: PLoS One. 2025 Nov 5;20(11):e0335409. doi: 10.1371/journal.pone.0335409 (PMC12588532; doi:10.1371/journal.pone.0335409)
Supplement: S3 Fig — (PDF) [file pone.0335409.s003.pdf]

Primary: Rabbit anti-pS199/202 Tau
Secondary: IR-Dye 800CW Goat anti-rabbit

Used in Fig S2B? X X X X X X X X X X X X

ILR2 No. 2 9 13 14 17 20 21 22 27 1 3 5 6 10 11 12 16 19 23 25 30 31 36 37

Group: AAV-IL-1RA AAV-GFP NonTg

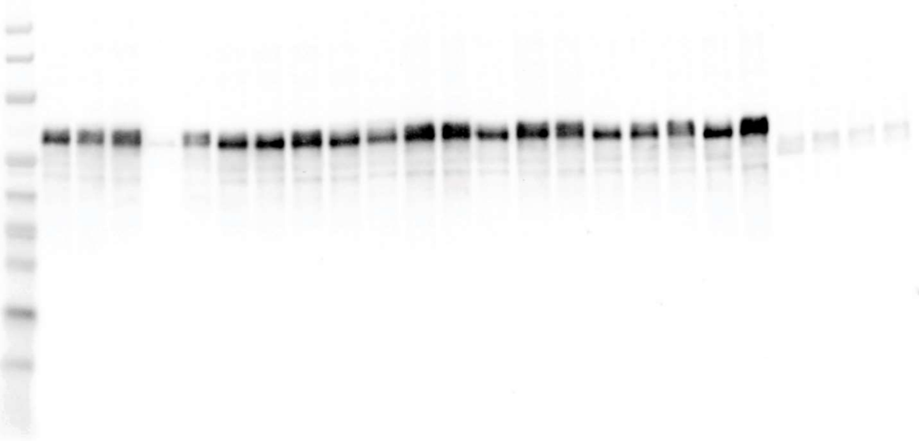

Bio-Rad TGX Stain-Free total protein stain

Used in Fig S2B? X X X X X X X X X X X X

ILR2 No. 2 9 13 14 17 20 21 22 27 1 3 5 6 10 11 12 16 19 23 25 30 31 36 37

Group: AAV-IL-1RA AAV-GFP NonTg

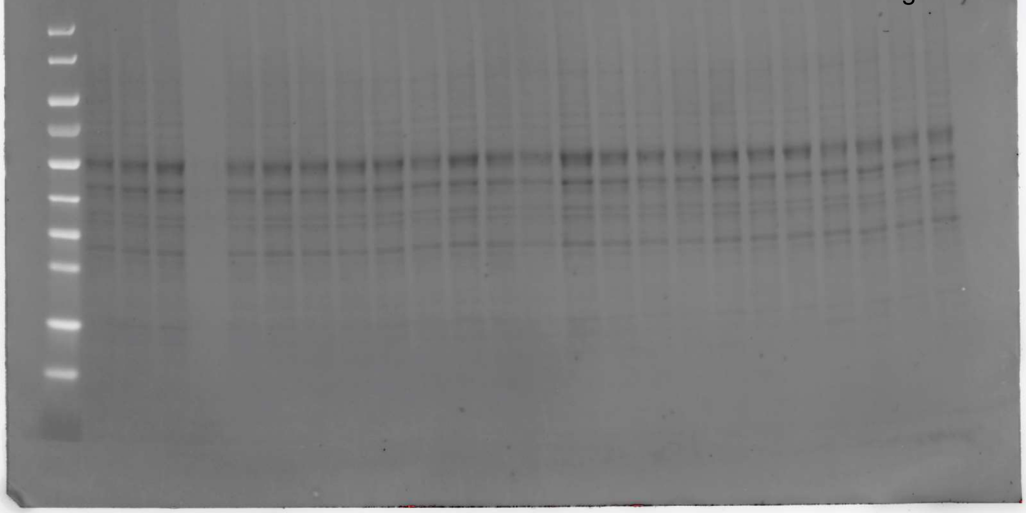

N.B.: ILR2-14 was not analyzed due to loading error.
