# Supplementary material for: Mature tau pathology is not improved by interfering with interleukin-1 receptor signaling in two mouse models of tauopathy
Source: PLoS One. 2025 Nov 5;20(11):e0335409. doi: 10.1371/journal.pone.0335409 (PMC12588532; doi:10.1371/journal.pone.0335409)
Supplement: Attachment — Submitted filename: Response to reviewers v4 082225.docx [file pone.0335409.s007.docx]

The authors would like to thank the reviewers for the cogent and thoughtful comments and helping to strengthen the manuscript. The authors would like to note that these two experiments were designed to test the hypothesis that using a gene therapy approach to over-express IL-1RA (recombinant IL-1RA is FDA-approved) would reduce tauopathy based on extensive literature in both mouse model systems and human studies implicating IL-1β in the progression of AD. We believe our data are timely and relevant to the current focus on inflammasome activation, which can increase secretion of IL-1β (among other actions). The goal of our experiment was to test this discrete, translational hypothesis, not to fully explore the biology of IL-1β or IL1R1 signaling in tauopathy. Our resulting data do not support the use of IL-1RA to diminish tauopathy. Although this does not directly contradict the possibility that IL-1 plays a role in Alzheimer’s pathogenesis, it would imply use of IL-1RA therapy is unlikely to benefit patients with tauopathy. Therefore, some of the suggested additional experiments we believe are beyond the scope of our conclusions. We have amended the revision to better align our conclusions with our hypothesis and data and to more fully discuss the limitations of our study design.

There were common concerns presented by both reviewers, which we would like to address here first. Both reviewers suggested measures *Il1b* message was insufficient and asked that we measure IL-1β protein in our model mice to confirm an increase. We have performed an ELISA for IL-1β in the soluble fraction of tissue homogenates of anterior cortices. We observed a significant 75% elevation of IL-1β in 6-month-old rTg4510s compared to 4 month-old nontransgenic mice (Figure 1B). Similarly, we observed small (10%) but significantly elevated IL-1β protein levels in 9-month-old PS19 mice compared to age-matched nontransgenic mice (Figure 6B). Published reports also detect modestly elevated IL-1β in rTg4510 mice by ELISA at 9 months (a trend was observed at 6 months) (Jiang et al., 2021) and PS19 mice at 4 months of age by IHC, preceding the development of mature tauopathy in PS19 mice (Yoshiyama et al., 2007). Using our IL-1β ELISA data, we were able to calculate the ratio of IL-1RA to IL-1β and we achieved approximately 70,000-fold and 1,000-fold excess IL-1RA in rTg4510s and PS19s, respectively. Thus, IL-1β protein is increased in our model systems and over-expression of IL-1RA by AAV yielded levels above the minimally effective IL-1RA to IL-1β ratio in both of our models.

Admittedly, we expected a greater induction of IL-1β by tauopathy based on reports from other inflammatory conditions. Recombinant IL-1RA is FDA approved for rheumatoid arthritis, in which IL-1β is increased by a factor of 2 in plasma (Eastgate et al., 1988) and a factor of 100 in synovial fluid (Kahle et al., 1992) compared to individuals without rheumatoid arthritis. In rTg4510s, Jiang et al. (2021) reports an approximately two-fold increase in IL-1β from 6-9mo, in line with our results. In Tau22 mice there is a reported 50% increase in IL-1β(p17) compared to nontransgenic controls (Ising et al., 2019). In an amyloid mouse model, Heneka et al. (2013) report an almost 3-fold increase in IL-1β compared to nontransgenic mice. However, in human AD brain there is an approximately 10-fold increase in IL-1β(p17) (Ising et al., 2019). Ghosh et al. (2013) reported IL-1 over-expression increased pTau in 3xTG mice, but these mice had a 9-fold increased expression of IL-1 over endogenous levels. It may be the case that amyloid pathology more strongly elicits increases in IL-1β than tauopathy, and this lower IL1β expression does not drive further tauopathy. We have added this information in the discussion

Additionally, both reviewers asked that we confirm our tau ELISA results using western blotting or some other method and to confirm that our tau transgenic mice do indeed have increased phospho-tau compared to nontransgenic mice. We did not run nontransgenic mice on tau ELISAs in our experiment involving rTg4510 mice, because in our 15 years of experience with the rTg4510 transgenic mouse line these mice have massively more phospho-tau and insoluble tau than nontransgenic littermate mice (Dickey et al., 2009; Lee et al., 2010; Brownlow et al., 2013; Nash et al., 2013; Selenica et al., 2013; Brownlow et al., 2014; Selenica et al., 2014a; Selenica et al., 2014b; Delic et al., 2015; Hunt et al., 2015; Joly-Amado et al., 2016; Finneran et al., 2019; Sandusky-Beltran et al., 2019; Joly-Amado et al., 2020). Additionally, the tau ELISA kits we purchased are advertised as using human-tau specific antibodies, (although some cross reactivity with mouse tau can be observed with high concentrations of human-tau antibodies such as AT8). Since we have less experience with the PS19 transgenic mouse line, we ran n=4 nontransgenic mice on each ELISA we performed in the study involving PS19 mice at equivalent dilutions to the transgenic mice and mouse tau was below the limit of detection in our nontransgenic samples. We have elected to use ELISA to measure tau pathology because the standard curves permit absolute quantitation (pg/mg) rather than relative concentration of the analyte (arbitrary units). Nonetheless, we have confirmed the pS199 ELISA data in this study by western with a similar antibody (pS199/202) and observed similar result that correlated well with the ELISA data (see supplemental figures). Additionally, we have also validated these ELISA kits by comparison to Western blots on a separate cohort of animals in a published study (Tetlow et al., 2023). Therefore, we believe our tau ELISA data to be reliable.

Below are our responses (in black) to specific reviewer comments (in red). We thank the reviewers again for their time spent reviewing our manuscript as well as their helpful comments in making this a stronger submission. We hope they find our responses satisfactory and look forward to any additional comments they may have.

Major comments:
2. While IL-1RA bioactivity was validated in vitro using a reporter cell line, the authors did not perform in vivo validation of IL-1β signaling inhibition. They could have challenged animals with LPS and assessed downstream inflammatory markers such as IL-6 to confirm effective blocking of IL-1β. Without such confirmation, it is unclear if the overexpression of IL-1RA seen in the mouse models resulted in inhibition of IL-1β in the brain.

The authors agree that a challenge experiment in which mice are administered AAV-GFP or AAV-IL1RA and then challenged with IL-1β (LPS has many other actions than increasing IL-1) ~4wks later would be a definitive demonstration of *in vivo* biological activity. However, this would require production of new AAV preparations, development of the mouse IL-1 injection model, and measurements of multiple outcome measures that would exceed the time restriction set for this revision. Additionally, the funding for this project is now expired and we are not able to undertake additional animal experiments. We have indicated this potential limitation of our study in the revised Discussion section but feel reasonably confident in the biological activity of the construct given the *in vitro* data and levels of *in vivo* expression of the protein.

3. The manuscript reports elevated Il1b mRNA in both models but does not assess levels of mature IL-1β protein, cleaved caspase-1, NLRP3 inflammasome, or downstream IL-1R signaling components such as NF-κB or MyD88. Without this information, whether IL-1β signaling is active at the ages they tested cannot be determined. It could be that IL-1β plays a more significant role at earlier stages of disease, before the intervention time points done in this study.

The authors thank the reviewer for this important question. Published reports contain increased protein measures of NLRP3, ASC, and cleaved caspase-1 in 6 month old rTg4510 mice (Jiang et al., 2021; de Oliveira et al., 2022). Similar results have been reported in PS19 mice at 3 and 6 months of age (Zhang et al., 2024). Coupled with our new data demonstrating increased IL-1β protein in our mice, we feel confident that IL-1β signaling is occurring in our model animals at the ages at which we applied our treatment. However, as indicated above, this level of increased signaling may not be sufficient to drive additional tau phosphorylation or deposition. This information has been added to the introduction section and our IL-1β ELISA results have been included in a revised figure in the manuscript.

4. To understand if IL-1RA over-expression had an impact, IL-1R1 expression levels and localization in the brain, authors are advised to refer to Liu X et al., (PMID: 30893590) in terms of normal physiological concentration (pM) vs pathological concentration (nM) of IL-1b. There could be two possibilities: a) low expression of IL-1R1 in cells other than endothelial and ependymal cells of the brain could explain the lack of response to IL-1RA (per PMID: 30893590) - that means, no point in having 300 or 100 fold over expression of IL-1RA when there is no receptor present in important cells (like microglia during early stages - until primed by endothelial cells?); b) 3 months (in rTg4510) and 6-months (PS19) time points may be already too late to block IL-1R signaling, which seem to originate in endothelial cells first. These points need to be discussed.

10. Finally, have the authors looked at the IL-1R1 levels in the brains of 3- and 6-month old rTg4510 and PS19 mice, respectively, in relation to non-transgenic controls as a first step before intervening with the IL-1RA?
The authors thank the reviewer for bringing this important study to their attention. We believe our approach is agnostic to which cell-types are expressing IL-1R1. Several studies have demonstrated that IL-1β is elevated in AD and mouse models of AD and that increased IL-1β can exacerbate AD-like pathology in mouse models. Therefore, our hypothesis was that antagonizing IL-1β signaling may be a potential therapeutic strategy in AD and was agnostic as to the precise biological mechanism(s) by which IL-1β may be exacerbating AD-like pathology. We have included the data from Liu et al in our discussion of IL1R1 biology in the Introduction section.

5. On a related note, the authors could have considered including two groups and starting IL-1RA both before and after the onset of tau pathology to understand whether IL-1R signaling plays a role in the initiation of disease versus its progression and assess the impact of IL-1RA on both. A short discussion on this limitation would also help.
6. If the authors hypothesize that tau pathology is independent of IL-1R activation, including IL-1β knockdown or knockout experiments in tauopathy models could help confirm this. Or highlighting these in the discussion is essential.

The authors agree that administering IL-1RA at a single timepoint in the disease, analogous to early in the disease state in humans, is a potential limitation of the study. However, we chose the time points for administering the AAV vectors to be relatively early such that either a role in prevention or in treatment of existing pathology could be detected. Furthermore, we agree that a more complete exploration of the role of IL-1β or IL1R1 signaling in these models would include genetic knockouts of both ligand and receptor. This is important future work that would meaningfully add to the discussion of inflammation in tauopathy and we have included it as a possible future direction in the Discussion section.

7. Including a brief explanation in the introduction or discussion about the IL-1R ligands (e.g., IL-1α, IL-1β, IL-1RA) and how IL-1RA competes with pro-inflammatory ligands for receptor binding would be helpful.

The authors agree that a brief discussion of IL1R1 biology is important context and we have added this to the Introduction section.

Minor comments
1. Fig. 1c and 1d – please specify what is measured in the Y axis (instead of ‘optical density’).

The Y-axis and figure legend has been altered for increased clarity.
2. Fig. 2b – Please provide statistics comparing contralateral groups (AAV-GFP vs AAV-IL-1RA).

All of the statistically significant comparisons are displayed on the graph. Although the two contralateral sides appear to be significantly different, this is likely be due to the use of the log scale Y-axis. The massive increases in IL-1RA in the ipsilateral treated group makes the variance of the overall experiment quite large. However, a direct T-test between the IL1-RA and GFP contralateral sides does demonstrate a significant difference. The figure has been updated to include a “ns” label for clarity.

Reviewer #2: In this study, the authors overexpressed interleukin-1 receptor antagonist (IL-1RA) in two different transgenic mouse models (rTG4510 and PS19) of tauopathy. However, the data showed that there were no significant changes in tauopathy after overexpression of IL- 1RA, so they concluded that interleukin-1 receptor signaling does not play a significant role in the progression of tauopathy in these two transgenic mouse models. Although it seems interesting, the experimental design lacks rigorous consideration, and the conclusion is not persuasive enough. Attached are some specific comments:

Please note the goal of the study was a test of IL-1RA gene therapy (see above), not a direct test of the IL-1 hypothesis. While the results do not support the IL-1 hypothesis, they are not sufficient to eliminate some form of the hypothesis. We have made this more clear throughout the manuscript.

2. Figure 2: The authors should include measuring IL-1β levels after the AAV-1L-1RA
treatment.

6. Figure 6: Still, how about the IL-1β signal levels change after overexpression of IL- 1RA
here? The author should include these measurements.

The authors agree this would be an interesting finding but unfortunately we do not have sufficient sample remaining to perform the measurements. We were able to measure the basal levels in the tau mice using additional samples of the same ages to establish IL-1 was increased, albeit by relatively small amounts (see above).

3. Figure 2C: The changes are not noticeable here. The authors should use a more
representative image.

The authors apologize for the confusion. This figure is IHC for GFP in both panels, which was used as a reporter for AAV-IL1-RA expression. These images are meant to demonstrate similar expression of both AAV constructs in mouse brain.

5. Figure5: The author should explain more about why soluble tau deposition correlated with IL1RA overexpression rather than insoluble tau deposition.

The authors apologize for the confusion. What is being correlated in these two figures are soluble total tau in the left hemisphere and soluble total tau in the right hemisphere, which are very tightly correlated, suggesting common causes for the interindividual animal variances (such as transgene copy number). However, insoluble total tau in the left hemisphere is not significantly correlated with insoluble total tau in the right hemisphere, indicating that deposition of tau may occur in a stochastic manner even within the same brain of an animal, with one side initiating deposition at a different time than the other.

8. Figure 10: The author should explain in detail why MCH-II’s expression is affected by the IL1RA.

We probed for MHC-II based on the results of our RNAseq experiment. Several of the differentially regulated genes are involved in antigen presentation and we wanted to confirm a reduction at the protein level. Reductions in these genes are broadly consistent with our predicted anti-inflammatory effect of IL-1RA and a reduction in MHC-II associated proteins has been observed after anti-inflammatory treatment by others in this mouse model (Yoshiyama et al.). We have made the rationale for this more clear in our Discussion section.

In Summary, we have been able to respond to some of the reviewer’s requests with new data, in some cases using archival samples from PS19 and rTg4510 mice. However, the additional animal experiments are beyond the scope of this manuscript as they would require 1) Generation of additional lots of AAV vectors 2) development of an animal model of IL-1 administration and 3) measurement of IL-1 responses within the time frame requested for the revision to be submitted. Additionally, there is no longer active funding for this project. We further have clarified that our hypothesis was the use of IL-1RA as a therapeutic in AD, based in part on the IL-1 hypothesis. We believe these data clearly contradict our original hypothesis that IL-1RA may be a useful therapeutic intervention to reduce tauopathy. However, while not supportive, we agree these data do not contradict the hypothesis that IL-1 has a role in AD pathogenesis, which we have stated more clearly in revision than in the original manuscript submission. It is feasible that the increases in IL-1 due to amyloid pathology may exceed those found in pure tauopathy models, and this may be sufficient to drive more extensive tauopathy. However, given the studies observing IL-1 can reduce Aβ deposition (Shaftel et al., 2007), and non IL-1 mechanisms by which inflammasome activation may promote AD (McManus et al., 2025), we suspect that human trials of IL-1RA to block tauopathy would not be a high priority for treating Alzheimer’s or other tauopathies at this time. We have extensively revised the Introduction and Discussion section as well as modified the existing figures to include our newly generated data in response to the reviewers’ comments.

**References:**

Brownlow, M.L., Benner, L., D'Agostino, D., Gordon, M.N., and Morgan, D. (2013). Ketogenic diet improves motor performance but not cognition in two mouse models of Alzheimer's pathology. *PLoS One* 8(9)**,** e75713. doi: 10.1371/journal.pone.0075713.

Brownlow, M.L., Joly-Amado, A., Azam, S., Elza, M., Selenica, M.L., Pappas, C., et al. (2014). Partial rescue of memory deficits induced by calorie restriction in a mouse model of tau deposition. *Behav Brain Res* 271**,** 79-88. doi: 10.1016/j.bbr.2014.06.001.

de Oliveira, P., Cella, C., Locker, N., Ravindran, K.K.G., Mendis, A., Wafford, K., et al. (2022). Improved Sleep, Memory, and Cellular Pathological Features of Tauopathy, Including the NLRP3 Inflammasome, after Chronic Administration of Trazodone in rTg4510 Mice. *J Neurosci* 42(16)**,** 3494-3509. doi: 10.1523/JNEUROSCI.2162-21.2022.

Delic, V., Brownlow, M., Joly-Amado, A., Zivkovic, S., Noble, K., Phan, T.A., et al. (2015). Calorie restriction does not restore brain mitochondrial function in P301L tau mice, but it does decrease mitochondrial F0F1-ATPase activity. *Mol Cell Neurosci* 67**,** 46-54. doi: 10.1016/j.mcn.2015.06.001.

Dickey, C., Kraft, C., Jinwal, U., Koren, J., Johnson, A., Anderson, L., et al. (2009). Aging analysis reveals slowed tau turnover and enhanced stress response in a mouse model of tauopathy. *Am J Pathol* 174(1)**,** 228-238. doi: 10.2353/ajpath.2009.080764.

Eastgate, J.A., Symons, J.A., Wood, N.C., Grinlinton, F.M., di Giovine, F.S., and Duff, G.W. (1988). Correlation of plasma interleukin 1 levels with disease activity in rheumatoid arthritis. *Lancet* 332(8613)**,** 706-709. doi: <https://doi.org/10.1016/S0140-6736(88)90185-7>.

Finneran, D.J., Morgan, D., Gordon, M.N., and Nash, K.R. (2019). CNS-Wide over Expression of Fractalkine Improves Cognitive Functioning in a Tauopathy Model. *J Neuroimmune Pharmacol* 14(2)**,** 312-325. doi: 10.1007/s11481-018-9822-5.

Ghosh, S., Wu, M.D., Shaftel, S.S., Kyrkanides, S., LaFerla, F.M., Olschowka, J.A., et al. (2013). Sustained interleukin-1beta overexpression exacerbates tau pathology despite reduced amyloid burden in an Alzheimer's mouse model. *J Neurosci* 33(11)**,** 5053-5064. doi: 10.1523/JNEUROSCI.4361-12.2013.

Heneka, M.T., Kummer, M.P., Stutz, A., Delekate, A., Schwartz, S., Vieira-Saecker, A., et al. (2013). NLRP3 is activated in Alzheimer's disease and contributes to pathology in APP/PS1 mice. *Nature* 493(7434)**,** 674-678. doi: 10.1038/nature11729.

Hunt, J.B., Jr., Nash, K.R., Placides, D., Moran, P., Selenica, M.L., Abuqalbeen, F., et al. (2015). Sustained Arginase 1 Expression Modulates Pathological Tau Deposits in a Mouse Model of Tauopathy. *J Neurosci* 35(44)**,** 14842-14860. doi: 10.1523/JNEUROSCI.3959-14.2015.

Ising, C., Venegas, C., Zhang, S., Scheiblich, H., Schmidt, S.V., Vieira-Saecker, A., et al. (2019). NLRP3 inflammasome activation drives tau pathology. *Nature* 575(7784)**,** 669-673. doi: 10.1038/s41586-019-1769-z.

Jiang, S., Maphis, N.M., Binder, J., Chisholm, D., Weston, L., Duran, W., et al. (2021). Proteopathic tau primes and activates interleukin-1beta via myeloid-cell-specific MyD88- and NLRP3-ASC-inflammasome pathway. *Cell Rep* 36(12)**,** 109720. doi: 10.1016/j.celrep.2021.109720.

Joly-Amado, A., Hunter, J., Quadri, Z., Zamudio, F., Rocha-Rangel, P.V., Chan, D., et al. (2020). CCL2 Overexpression in the Brain Promotes Glial Activation and Accelerates Tau Pathology in a Mouse Model of Tauopathy. *Front Immunol* 11**,** 997. doi: 10.3389/fimmu.2020.00997.

Joly-Amado, A., Serraneau, K.S., Brownlow, M., Marin de Evsikova, C., Speakman, J.R., Gordon, M.N., et al. (2016). Metabolic changes over the course of aging in a mouse model of tau deposition. *Neurobiol Aging* 44**,** 62-73. doi: 10.1016/j.neurobiolaging.2016.04.013.

Kahle, P., Saal, J.G., Schaudt, K., Zacher, J., Fritz, P., and Pawelec, G. (1992). Determination of cytokines in synovial fluids: correlation with diagnosis and histomorphological characteristics of synovial tissue. *Ann Rheum Dis* 51(6)**,** 731-734. doi: 10.1136/ard.51.6.731.

Lee, D.C., Rizer, J., Selenica, M.L., Reid, P., Kraft, C., Johnson, A., et al. (2010). LPS- induced inflammation exacerbates phospho-tau pathology in rTg4510 mice. *J Neuroinflammation* 7(56)**,** 56. doi: 10.1186/1742-2094-7-56.

McManus, R.M., Komes, M.P., Griep, A., Santarelli, F., Schwartz, S., Ramon Perea, J., et al. (2025). NLRP3-mediated glutaminolysis controls microglial phagocytosis to promote Alzheimer's disease progression. *Immunity* 58(2)**,** 326-343 e311. doi: 10.1016/j.immuni.2025.01.007.

Nash, K.R., Lee, D.C., Hunt, J.B., Jr., Morganti, J.M., Selenica, M.L., Moran, P., et al. (2013). Fractalkine overexpression suppresses tau pathology in a mouse model of tauopathy. *Neurobiol Aging* 34(6)**,** 1540-1548. doi: 10.1016/j.neurobiolaging.2012.12.011.

Sandusky-Beltran, L.A., Kovalenko, A., Ma, C., Calahatian, J.I.T., Placides, D.S., Watler, M.D., et al. (2019). Spermidine/spermine-N(1)-acetyltransferase ablation impacts tauopathy-induced polyamine stress response. *Alzheimers Res Ther* 11(1)**,** 58. doi: 10.1186/s13195-019-0507-y.

Selenica, M.B., Benner, L., Housley, S.B., Manchec, B., Lee, D.C., Nash, K.R., et al. (2014a). Histone deacetylase 6 inhibition improves memory and reduces total tau levels in a mouse model of tau deposition. *Alz Res Therapy* 6(12). doi: <https://doi.org/10.1186/alzrt241>.

Selenica, M.B., Davtyan, H., Housley, S.B., Blair, L.J., Gillies, A., Nordhues, B.A., et al. (2014b). Epitope analysis following active immunization with tau proteins reveals immunogens implicated in tau pathogenesis. *J Neuroinflammation* 11(152). doi: <https://doi.org/10.1186/s12974-014-0152-0>.

Selenica, M.L., Brownlow, M., Jimenez, J.P., Lee, D.C., Pena, G., Dickey, C.A., et al. (2013). Amyloid oligomers exacerbate tau pathology in a mouse model of tauopathy. *Neurodegener Dis* 11(4)**,** 165-181. doi: 10.1159/000337230.

Shaftel, S.S., Kyrkanides, S., Olschowka, J.A., Miller, J.N., Johnson, R.E., and O'Banion, M.K. (2007). Sustained hippocampal IL-1 beta overexpression mediates chronic neuroinflammation and ameliorates Alzheimer plaque pathology. *J Clin Invest* 117(6)**,** 1595-1604. doi: 10.1172/JCI31450.

Tetlow, A.M., Jackman, B.M., Alhadidy, M.M., Muskus, P., Morgan, D.G., and Gordon, M.N. (2023). Neural atrophy produced by AAV tau injections into hippocampus and anterior cortex of middle-aged mice. *Neurobiol Aging* 124**,** 39-50. doi: 10.1016/j.neurobiolaging.2022.06.014.

Yoshiyama, Y., Higuchi, M., Zhang, B., Huang, S.M., Iwata, N., Saido, T.C., et al. (2007). Synapse loss and microglial activation precede tangles in a P301S tauopathy mouse model. *Neuron* 53(3)**,** 337-351. doi: 10.1016/j.neuron.2007.01.010.

Zhang, L., Gai, Y., Liu, Y., Meng, D., Zeng, Y., Luo, Y., et al. (2024). Tau induces inflammasome activation and microgliosis through acetylating NLRP3. *Clin Transl Med* 14(3)**,** e1623. doi: 10.1002/ctm2.1623.
