# Supplementary material for: Mature tau pathology is not improved by interfering with interleukin-1 receptor signaling in two mouse models of tauopathy
Source: PLoS One. 2025 Nov 5;20(11):e0335409. doi: 10.1371/journal.pone.0335409 (PMC12588532; doi:10.1371/journal.pone.0335409)
Supplement: Attachment — Submitted filename: comment on IL manuscript.docx [file pone.0335409.s006.docx]

The authors have concluded interleukin-1 receptor signaling does not play a significant role in the progression of tauopathy using two transgenic models rTg4510 and PS19 models. Both these models does not inherently manifest amyloid-beta (Aβ) pathology; it primarily develops tau pathology, such as neurofibrillary tangles, mimicking human tauopathies. Previously it has been investigated by various researcheres the role of IL-1β in AD pathogenesis, using inducible model of sustained IL-1β overexpression using 3xTg-AD model, tau phosphorylation despite an ∼70–80% reduction in amyloid load and fourfold to sixfold increase in plaque-associated microglia, as well as evidence of greater microglial activation at the site of inflammation. There was also evidence of increased p38 mitogen-activated protein kinase and glycogen synthase kinase-3β activity, which are believed to contribute to tau phosphorylation.

Can the authors comment on that what relvance they can emphsize on their studies. Also does the authors looked over to to pathways like regulation of p38 mitogen-activated protein kinase and glycogen synthase kinase-3β activity .
