# Supplementary material for: Mature tau pathology is not improved by interfering with interleukin-1 receptor signaling in two mouse models of tauopathy
Source: PLoS One. 2025 Nov 5;20(11):e0335409. doi: 10.1371/journal.pone.0335409 (PMC12588532; doi:10.1371/journal.pone.0335409)
Supplement: Attachment — Submitted filename: IL1RA resub2 Response to Editor Oct2025.docx [file pone.0335409.s008.docx]

Revision of **Mature tau pathology is not improved by interfering with interleukin-1 receptor signaling in two mouse models of tauopathy**

Dear Dr Rasool,

In your response to our revision, you mention the publication by Ghosh et al (2013) examining the role of IL-1 overexpression on tau pathology in the 3xTg mouse model (a paper we have referenced here and on multiple other occasions). The data presented in Ghosh and a few other publications led to our hypothesis that treatment with an FDA approved drug, Anakinra (human IL-1RA) might reduce tauopathy including that in AD. Prior work found elevated IL-1β RNA in the pure tauopathy models used here, and we assumed they would be useful models to test the role of IL-1RA gene therapy as a possible therapy for tauopathies. In spite of achieving high levels of IL-1RA, we failed to detect any impact on tau pathology in these mice. We then went to measure the levels of IL-1β protein in these mice and found relatively small elevations. Thus, we conclude that IL-1β is unlikely to be driving tau pathology in these mice and this explains why the IL-1RA gene therapy had no effects on tauopathy.

Perhaps had we had a mouse with both amyloid and tau, we would have found a different outcome, but our recent experience with the 3xTg model found no tauopathy in aged mice as the mouse has lost phenotype over time (this is also stated to be the case on the JAX web site).

For the most part this was indicated in the paper discussion, but the abstract was still focusing on the role in AD, which we now agree was not fully addressed in the pure tauopathy models. We have rewritten the abstract to more clearly explain the conclusions we have reached. However, for the pure tauopathies, we believe the manuscript explains that IL-1β is unlikely to be driving tauopathy in these models, and thus IL-1RA gene therapy is unlikely to have therapeutic value.

With respect to your second comment, regarding possible alterations in tau kinase regulation due to IL-1RA over-expression, we did not investigate this possibility because we did not observe changes in phospho-tau levels at sites known to be phosphorylated by these kinases. While it is possible that there are changes in the activity of one of these kinases and compensatory increases in activity of the other, our initial hypothesis was that IL-1RA over-expression would reduce tau phosphorylation. Finding that the data did not support this hypothesis, we did not pursue mechanism further.

We now include the revised manuscript, both with and without mark up, indicating the changes in the abstract and in a few places of the discussion.
